# A new mouse model of ankle instability induced by multiple mechanical sprains with controlled inversion angle and speed

**DOI:** 10.3389/fbioe.2022.927987

**Published:** 2022-09-02

**Authors:** Shih-Hong Ching, Yen-Chun Chiu, Yu-Ching Liao, Shang-Hsun Yang, Yi-Ju Tsai

**Affiliations:** ^1^ Institute of Basic Medical Science, College of Medicine, National Cheng Kung University, Tainan, Taiwan; ^2^ Department of Orthopaedic Surgery, E-Da Hospital, I-Shou University, Kaohsiung, Taiwan; ^3^ Department of Physiology, College of Medicine, National Cheng Kung University, Tainan, Taiwan; ^4^ Department of Physical Therapy, College of Medicine, National Cheng Kung University, Tainan, Taiwan; ^5^ Institute of Allied Health Sciences, College of Medicine, National Cheng Kung University, Tainan, Taiwan

**Keywords:** lateral ankle sprain, ligamentous injury, animal model, chronic ankle instability, gait

## Abstract

Ankle sprain occurs by a sudden and extreme inversion and plantarflexion at the ankle joint to cause ligamentous injuries. A portion of ankle sprain patients experience recurrent ankle sprains and develop chronic ankle instability (CAI). The present CAI animal models are single events with severe ligamentous injury using surgical transection of ligaments or manually overextending the ankle.

**Purpose:** To simulate the mechanical and recurrent sprain injuries in CAI patients, we established a new ankle instability model with multiple ankle injuries using a self-designed machine to sprain the ankle with a controlled inversion angle and speed.

**Methods:** Male C57BL/6J mice were used and respectively subjected to a sham operation, calcaneofibular ligament (CFL) transection, and mechanical ankle sprains. Three mechanical sprains were performed on the 13th and 185th day after the initial mechanical ankle sprain.

**Results:** The first mechanical sprain and CFL transection induced ankle injury as indicated by an average of a 62% decrease in ankle pressure pain threshold and a 114% increase in the ankle thickness compared with the contralateral untreated ankle. The second and third mechanical sprains induced recurrent ankle injuries. The foot slips during beam tests were increased after mechanical ankle sprains but not after CFL transection, indicating the induction of motor balance deficits. Multiple mechanical ankle sprains induced significant gait changes in longer duration of stance (an average of 194% increase), swing (134%), and step cycle (147%) compared with CFL transection or sham operation, and slower walking speed (78% reduction) and shorter step distance (91%) after the third sprain.

**Conclusion:** These results elucidate that multiple mechanical sprains, which induce recurrent ankle injuries, balance deficits, and gait changes, are a good model for investigating the mechanisms of CAI induced by recurrent sprain injuries.

## Introduction

Ankle sprain is the most common sports injury, with a rate of 4.95 lateral ligament complex ankle sprains per 10,000 athlete-exposures (Roos et al., 2017). Lateral ankle sprain is the most common type among all ankle sprains (Garrick and Schelkun, 1997). When the ankle experiences an inversion and/or plantarflexion motion that exceeds physiological limits, ligaments on the lateral side of the ankle are often damaged, including the anterior talofibular ligament (ATFL), posterior talofibular ligament (PTFL), and calcaneofibular ligament (CFL). The severity of the lateral ankle sprain is often classified into grades I (mild), II (moderate), and III (severe), respectively, representing only stretching, partial, and complete rupture of one or more lateral ligaments (Tiemstra, 2012; Slater, 2018). An acute ankle sprain could result in pain, swelling, and loss of function. Residual symptoms after an acute ankle sprain affect 55%–72% of patients at 6–18 months (Gerber et al., 1998; Braun, 1999).

Although conservative treatments often lead to full functional recovery in most people, up to 26% of the patients continue to have recurrent ankle sprains, suggesting chronic instability of the ankle joint. Chronic ankle instability (CAI) is defined as the patient having recurrent lateral ankle sprains after the initial sprain and is characterized by a recurring giving way sensation (i.e., instability) of the ankle during walking or other activities (Hertel, 2002). Depending on different populations, up to 70% of patients suffering from lateral ankle sprain develop CAI (Fong et al., 2007; Swenson et al., 2009; Hiller et al., 2012). CAI has also been found to have a higher risk of developing post-traumatic osteoarthritis (Gribble et al., 2016; Liu et al., 2021; Li et al., 2022).

The common symptoms of CAI include balance deficits, decreased physical activity (Wikstrom et al., 2018), and recurrent injuries (Yeung et al., 1994). CAI includes mechanical instability, functional instability, or both. Mechanical instability is the measurable anatomical change, such as joint laxity, that causes ankle joint motion to exceed normal physiological limits (Tropp, 2002). Functional instability is the sensation of ankle joint instability and repetitive re-injuries. The impairment of proprioception, neuromuscular control, postural control, and strength are proposed to be involved in functional instability. However, the supporting evidence is still poor (Hertel, 2000; Tropp, 2002). Therefore, an appropriate animal model is urgently needed to study the underlying mechanisms and treatments for ankle instability.

Currently, two ankle sprain models have been developed. The first one is a mouse model by surgically transecting the CFL and ATFL (Kim et al., 2008; Kim et al., 2011; Hubbard-Turner et al., 2013; Wikstrom et al., 2015; Liu et al., 2021; Li et al., 2022). The second one is a rat model induced by manually overextending the ankle repeatedly in the plantarflexion and inversion directions (Koo et al., 2002). The ligament transection model is a single injury event with severe injury, which does not replicate the recurrent sprains seen in the CAI patients. The manual ankle sprain model was developed for investigating ankle sprain related pain. It is still unknown whether other CAI-related symptoms can be induced by this model or not (Koo et al., 2008). Furthermore, twisting the ankle manually may introduce inter-experimenter variability, while twisting the ankle joint 60 times in 1 minute does not match how ankle sprain injuries occur in humans. However, these two models do not fulfil the development of CAI by recurrent and mechanical sprains in humans and may also not be appropriate for studying whether incomplete ligamentous (mild and moderate) injuries are able to induce CAI.

In order to simulate mechanical and recurrent inversion sprains seen in CAI patients, it is crucial to develop a non-invasive (closed) lateral ankle sprain animal model that replicates the ankle sprain motion more faithfully, with lower intra- and inter-experimenter variations. We designed a machine that has a computer-controlled motor capable of rotating the mouse ankle joint in the inversion direction with precise control of rotation angle and speed. Using the self-designed machine, we developed a new mouse model of mechanical ankle sprain and demonstrated that two consecutive twists (117° of inversion at the speed of 1,500° per second) can induce an acute ankle sprain injury similar to that induced by CFL transection. We also further performed multiple sprains to investigate whether CAI could be induced.

## Materials and methods

### Animals

All experimental protocols were in accordance with the National Institutes of Health Guidelines for Animal Research and were approved by the National Cheng Kung University Institutional Animal Care and Use Committee (IACUC #109126). All efforts were made to ensure animal welfare. Male 8-week old C57BL/6J mice (body weight 18–22 g) were used and obtained from the animal facility of the Medical College, National Cheng Kung University, Tainan, Taiwan. The mice were housed in a 12/12-h light/dark cycle room at 22 ± 2°C and had free access to standard laboratory chow and water *ad libitum*. Twelve mice were divided into three groups, *n* = 4 for each group: the sham operation group, the CFL transection group, and the mechanical sprain group. Mice were randomly allocated into groups after the baseline measures were made.

### Surgical injury model

For both the sham operation and CFL transection groups, mice were anesthetized in a chamber using 4% isoflurane and kept under anesthetization using a mask. Prior to the surgery operation, the lateral side of the right ankle was shaved and cleaned with 75% alcohol for both groups. A small incision was made longitudinally on the anterolateral side of the right ankle. Under the anatomical microscope, the CFL was transected using a purpose-made hook tool with sharpened edges (Kim et al., 2008; Wikstrom et al., 2018). After the operation was finished, the wound was sutured with polyamide monofilament (6/0, B. Braun Surgical, S.A. Rubi, Spain). Mice received subcutaneous injection of 5.0 mg·kg-1 Carprofen (Rimadyl; Zoetis, Parsippany, NJ), one injection per day for 3 days to prevent wound inflammation. For the sham operation group, the mice were anesthetized following the same procedure as the CFL transection group. The same incision as the CFL transection group was made, but no other surgical operations were performed. After the incision, the wound was sutured with the same procedure as the CFL transection group, following the same wound care protocol.

### Mechanical sprain model

**FIGURE 1:**
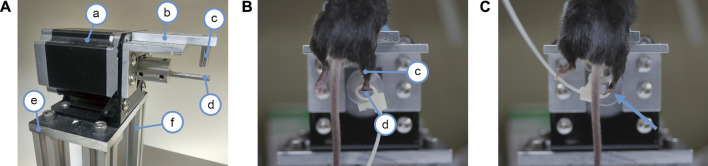
Device for mechanical ankle sprain. **(A)** photograph showing the device for spraining the ankle, a: a stepper motor, b: bed platform for the mouse, c: bed post for restraining the mouse shin to the machine, d: foot rest rod connected to the motor shaft for securing mouse foot to the motor, e: base plate to mount the motor, f: posts to support the motor. **(B)** photograph showing a mouse mounted on the ankle spraining machine. The right shin is held against the bed post (c) by the experimenter’s hand (not shown) to restrict shin movement. The right paw is secured on the foot rest rod (d) by a cable tie. In photo **(B)**, the mouse foot is parallel to the floor and the mouse ankle has not yet been rotated. **(C)** photograph showing how much the motor shaft spins to sprain the ankle, it spins 117° in the inversion direction as indicated by an arrow.

To establish a non-invasive (closed) lateral ankle sprain model, a device was designed and fabricated (Figure 1) that can restrain the mouse securely and precisely rotate the ankle joint in the inversion/eversion axis. A stepper motor (Figure 1Aa) is bolted on a heavy base (Figure 1Ae). The motor is powered by a 200W power supply and controlled by a computer-governed controller. A rod (Figure 1Ad) coupled to the motor shaft acted as the foot rest for securing the mouse foot in place. The rod was cut in half length-wise, thus, when the foot was resting on the foot rest, the rotation axis of the motor was able to come in line with the inversion/eversion axis of the ankle. A plate (Figure 1Ab) affixed on top of the motor acted as the bed platform to hold the mouse body. Above the foot rest rod, a bed post (Figure 1Ac) was affixed on the underside of the bed platform to provide an anchor point for restraining the mouse shin to the machine.

Mice were anaesthetized with inhalation of 4% isoflurane and then mounted on the bed platform, facing downwards. A cable tie was used to strap the mouse paw to the rod (foot rest) connected to the motor shaft. The mouse shin was held against the bed post by the experimenter’s hand to restrict the shin’s movement. After the mouse was strapped firmly in place, the right ankle joint was inverted in the inversion/eversion axis two times. A video was provided to demonstrate the condition of the mechanical sprain (Supplementary Video S1). The ankle was rotated to 117° of inversion at a speed of 1,500° per second. Based on previous studies of human ankle sprain kinematics (Fong et al., 2009; Kristianslund et al., 2011; Fong et al., 2012), we decided on a rotation speed of 1,500° per second. Our preliminary cadaver trials found that the mice have a much greater inversion range (an average of 90°) compared to humans (30°) (Norkin and White, 2016). To ensure that the mechanical sprains exceed the physiological limit of the ankle joint and accommodate the characteristics of the stepper motor of the device, 117° of inversion rotation was chosen for our study.

### The measurement for ankle pain threshold

Pain at the joint site is one of the symptoms of ankle sprain. Pressure application measurement (PAM) was used to measure the ankle joint hypersensitivity to pressure (Barton et al., 2007). A 3 kg capacity load cell was used to measure how much force was applied. A load cell with a stainless rod (5 mm diameter) attached, acting as pressure point, was used to assert pressure on the mice’s ankle. Mice were accustomed to being handled and restrained before the experiments. The intraperitoneal injection position was suited for measuring ankle joint hypersensitivity since the hind limbs were easily accessible. Following the measuring procedure provided by Barton et al. (2007), the load cell was used to apply force on the lateral side of the ankle joint across the joint with gradually increasing force. When mice showed withdrawal response with their legs or squealed, the load cell was released and the maximal force was recorded. Each mouse was measured three times at each time point and the average was shown.

### The measurement for ankle swelling

Swelling at the ankle joint is another one of the primary symptoms of an ankle sprain. A handheld thickness gauge was used to measure the level of ankle swelling. Mice were trained to be handled and restrained before the experiment started. Each ankle joint was measured three times at each time point.

### Beam test

The beam test is a commonly used test for assessing movement coordination (Luong et al., 2011). In brief, the beam is a transparent acrylic plate 1 m in length, 6 mm in width, 20 mm in height, and suspended 50 cm above the floor. A camera was used to capture footages of mice traversing the beam. Mice were trained for 3 days to get accustomed to the beam test before the experiment started. Mice traversed the beam three times for each time point. The speed of mice traversing the beam and the total number of hind foot slips were counted by two experimenters who were blind to the grouping.

### Footprint assay

The footprint assay is based on the optical phenomenon of frustrated total internal reflection (FTIR) as previously described (Mendes et al., 2015). In brief, the footprint apparatus consists of a walkway, lights, a mirror, and a camera. The floor of the walkway was a 10 mm thick transparent acrylic panel. Two strips of blue light-emitting diodes (LEDs) were affixed to both sides of the floor acrylic to provide the light source for the FTIR. A mirror was located 45° underneath the floor panel to reflect the images of the underside of the floor panel towards the camera, which recorded the footage of mice’s footprints traversing the walkway. The footprint assay was performed three times at each time point.

Recorded footages were loaded into the footprint analyzing program developed by Mendes et al. (2015). The program is capable of identifying footprint locations automatically and footprint parameters such as stance duration, swing duration, step cycle, step distance, foot size, and overall speed can be extracted. Since only the right hind limb was injured, the performance of the hind limbs was analyzed. Several gait cycles were recorded when a mouse traversed the walkway, and the average of each gait cycle was calculated. Stance duration was the duration of the stance phase of the gait cycle, while the swing duration was the the duration of swing phase of the gait cycle. A step cycle is the duration of each gait cycle. Step distance was the distance between the centroids of two sequential steps of the same paw. Foot size is the size of the lightened up area on the walkway for each paw. Overall speed is obtained from dividing the walkway length by the time the mouse takes to finish it, and can be treated as walking speed.

### Statistical analysis

The data were analyzed and drawn in the GraphPad Prism 9.3 (GraphPad Software, San Diego, CA, United States), and represented as mean± standard error of means (S.E.M). The statistical significance was analyzed using a paired *t* test within the group, such as a comparison between the left untreated foot and the right treated foot, or a comparison before (baseline) and after the treatment. The statistical significance was analyzed using an unpaired *t* test between the independent groups, including a comparison between the sham group and the surgery group, or the sham group and the machine group, or the surgery group and the machine group.

## Results

### Mechanical ankle sprain induces pain on ankle

**FIGURE 2:**
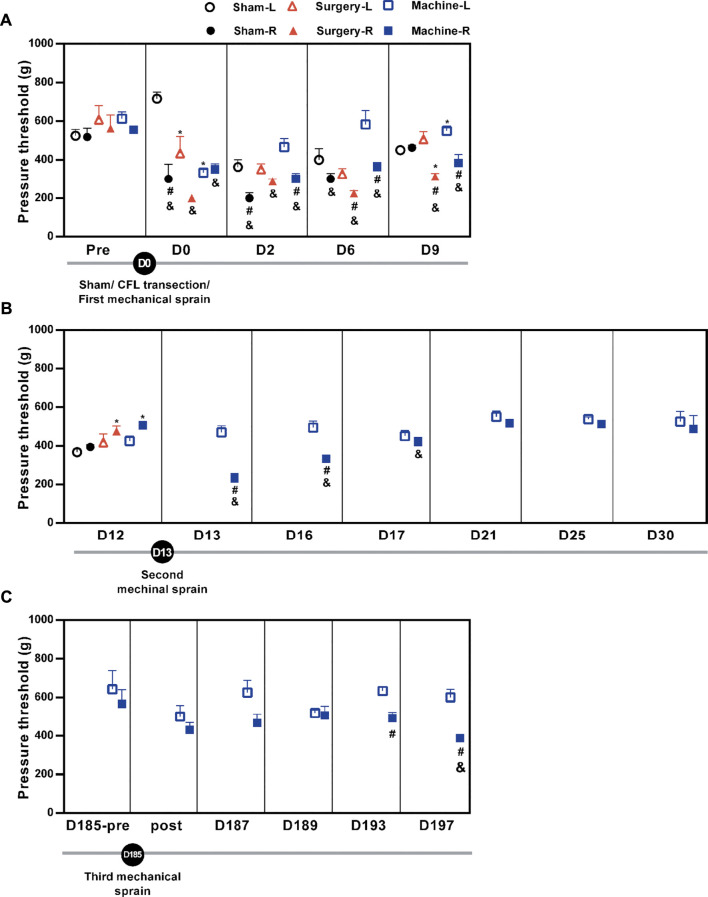
Changes in the pressure pain threshold after mechanical ankle sprains. The ankle pain threshold was measured using PAM on the indicated day. The pain threshold is shown **(A)** after the sham operation or the CFL transection or the first mechanical ankle sprain on day 0 (D0), **(B)** after the second mechanical ankle sprain on the 13th day (D13), and **(C)** before (D185-pre) and after (post) the third mechanical ankle sprain on the 185th day (D185). Values are mean ± SEM, *n* = 4. The statistical significance was analyzed using paired *t* test within the group (# and &) or unpaired *t* test between groups (*). *p < 0.05 compared with corresponding sham group; #*p* < 0.05 compared with corresponding left untreated foot (L); & *p* < 0.05 compared with the corresponding right treated foot (R) of pretreatment (pre) baseline on the 12th day (D12) in **(B)** and pretreatment baseline on the 185th day (D185-pre) in **(C)**.

Hypersensitivity to pressure (pain) is a major indicator for ankle sprain injury. We evaluated whether the ankle sprain under the condition of 117° of inversion rotation and at a speed of 1,500° per second could induce pressure hypersensitivity on the injured ankle using PAM. Results showed that pressure threshold to induce foot withdrawal was decreased, indicating induction of pain on the injured ankles in all groups, with each group sustaining a different duration of decrease. (Figure 2A). The pressure threshold of the right treated foot was significantly reduced after the CFL transection (an average of 65 ± 3% reduction for D6–D9 or 42 ± 5% for D0–D9) and also after the first mechanical sprain (an average of 62 ± 5% for D3–D9 or 63 ± 3% for D0–D9), respectively, compared with the contralateral (untreated) left foot or corresponding pretreatment baseline on the right foot (Figure 2A). These results demonstrated that the induction of pressure hypersensitivity by a single mechanical sprain was similar to that induced by CFL transection. In the sham operation group, decreased pressure threshold was found only immediately (D0) and on the second day (D2) after the sham operation. Moreover, results showed that pressure hypersensitivity on the injured side reappeared on the 13th day (D13, 51% reduction of pressure threshold vs. the contralateral left foot and 46% vs. the right foot of baseline on the D12), D16 (67% and 66%), and D17 (93% and 83%, Figure 2B). The third mechanical sprain also induced a significant decrease in pressure threshold on the 193th (78% reduction vs. the contralateral left foot and 87% vs. the injured right foot of baseline on the D185-pre) and 197th day (65% and 68%, Figure 2C). These results showed that a single mechanical ankle sprain is successful in inducing a similar pressure hypersensitivity caused by surgical CFL transection and that recurrent ankle sprain injuries can be developed by multiple mechanical sprains.

### Swollen ankle is found after mechanical ankle sprain

**FIGURE 3:**
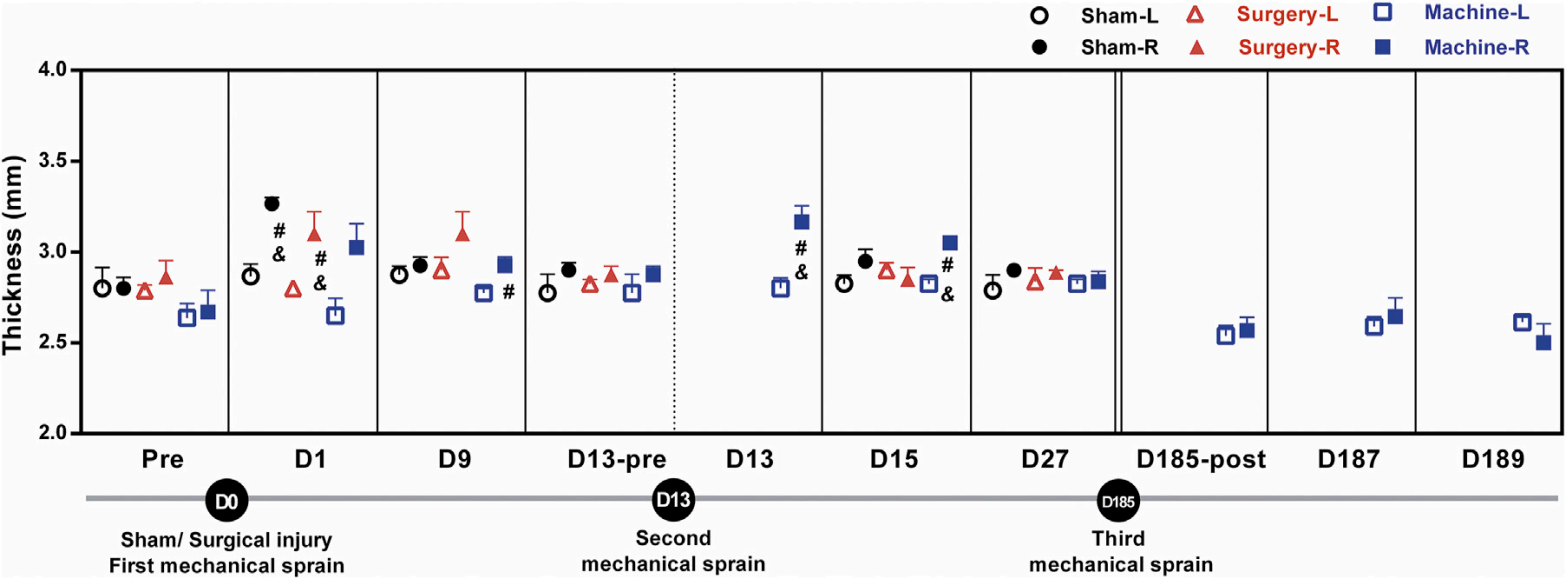
Changes in ankle thickness after mechanical ankle sprains. The level of ankle swelling was measured using a thickness gauge on the indicated day after sham operation or CFL transection or three times of mechanical ankle sprains. The statistical significance was analyzed using paired *t* test, #*p* < 0.05 compared with corresponding left untreated foot (L) and & *p* < 0.05 compared with the corresponding right foot (R) of pretreatment (pre) baseline or before the second mechanical sprain (D13-pre) on the 13th day (D13).

One of the major symptoms of ankle sprain-induced inflammation is ankle swelling; the swelling was evaluated by measuring the ankle thickness with a thickness gauge. Results showed that ankle thickness in the treated right side increased significantly on the 1st day (D1) only after the sham operation (3.27 ± 0.03 mm) and CFL transection groups (3.2 ± 0.08 mm), respectively, compared with the contralateral uninjured side (2.87 ± 0.06 and 2.8 ± 0.00), or compared to the injured side on the baseline before the treatment (2.8 ± 0.06 and 2.86 ± 0.09, Figure 3). The first mechanical sprain induced an increasing trend in ankle thickness on D1 (3.03 ± 0.13 on the sprained ankle vs. 2.65 ± 0.09 on the un-sprained ankle, *p* = 0.14) and a significant increase on D9 (2.93 ± 0.04 on the sprained ankle vs. 2.77 ± 0.02 on the un-sprained ankle). These results indicated that in comparison with an acute ankle swelling induced by CFL transection, a single mechanical sprain induces a prolonged effect on the ankle swelling. Moreover, a swollen ankle reoccurred after the second mechanical sprain as shown by a significant increase in sprained ankle thickness compared to the un-sprained side (3.16 ± 0.07 mm vs. 2.80 ± 0.06 mm on the D13 and 3.05 ± 0.03 mm vs. 2.82 ± 0.03 mm on the D15) or compared to the injured side before the second sprain (2.87 ± 0.05 mm). However, the third mechanical sprain on the 185th day did not induce a significant increase in the thickness of the sprained ankle. The results demonstrated that in contrast to the one-time injury induced by CFL transection, the repeated mechanical sprains within 2 weeks were able to induce a recurrent ankle swelling but could not reproduce the swelling symptoms 6 months after the initial sprain.

### Mechanical ankle sprain induces impairment of motor coordination

**FIGURE 4:**
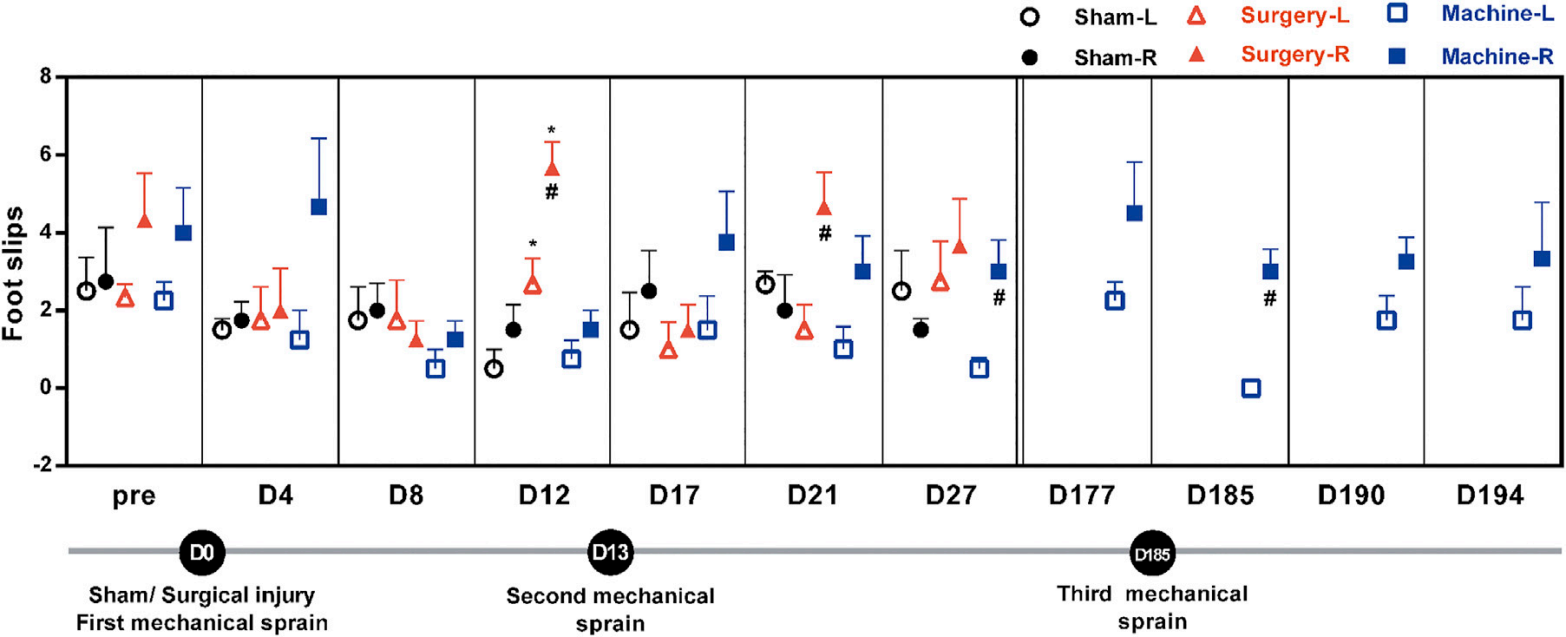
Effect of mechanical ankle sprains on motor balance and coordination. The number of hind foot slips were measured using a beam test on the indicated day to evaluate motor balance and coordination after sham operation, CFL transection, and three mechanical sprains. Total number of foot slips were counted in three tests at each time point. Values are mean ± SEM, *n* = 4. #*p* < 0.05 compared with corresponding left untreated foot (L) using paired *t* test. *p < 0.05 compared with sham operation group using unpaired *t* test.

We performed the beam test to examine motor coordination after repeated mechanical ankle sprains. Results showed that after the first mechanical ankle sprain, the number of foot slips had a brief upward trend on the sprained side (4.67 ± 1.53 vs. 1.25 ± 0.75 on the un-sprained side, *p* = 0.18) on the 4th day. The sham operation did not induce an increase in foot slips on the treated side. CFL transection induced a significant increase in foot slips of the injured side compared with the un-injured side (5.67 ± 0.57 vs. 2.66 ± 0.66 on the D12 and 4.66 ± 0.76 vs. 1.5 ± 0.64 on the D21, Figure 4). After the second mechanical sprain on the 13th day (D13), an increasing trend of foot slips was persistently found from D17 to D177, with a significantly higher number of slips on the 27th day (3.67 ± 0.57 vs. 0.33 ± 0.28 on D27) compared to the un-sprained side. Moreover, after the third mechanical sprain, a significantly higher number of foot slips on the sprained side was reproduced (3 ± 0.5 vs. 0 ± 0 on the un-sprained side) and continually found until the end of the experiment. These results demonstrated that repeated mechanical ankle sprains induced a long-term balance deficit.

### Abnormal gait parameters are found after mechanical ankle sprain

**FIGURE 5:**
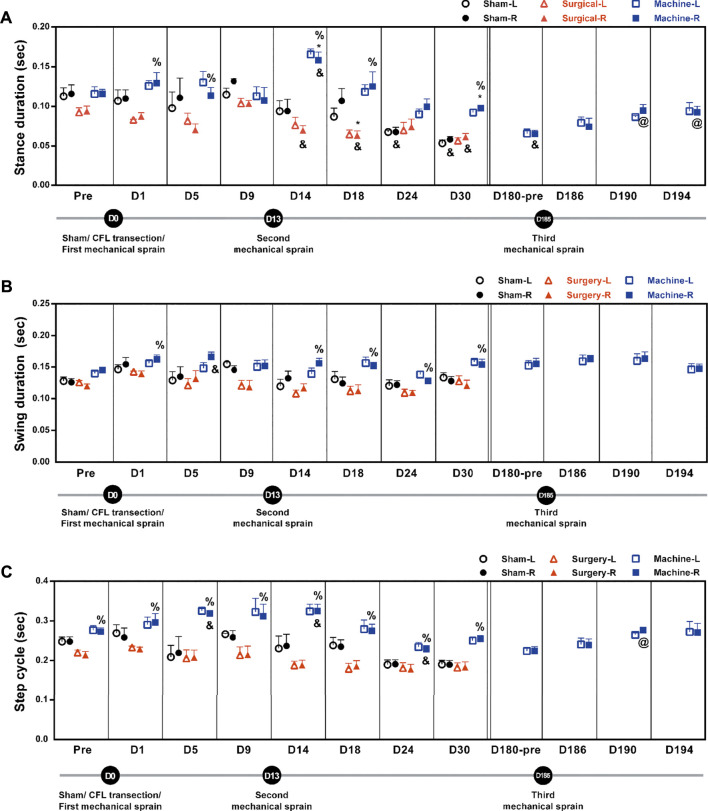
Mechanical ankle sprains induces gait abnormalities in stance duration, swing duration, and step cycle. Footprint was measured and analyzed on days indicated on the graphs after sham operation, CFL transection, and three mechanical ankle sprains to obtain gait parameters including stance duration **(A)**, swing duration **(B)** and step cycle **(C)**. Values are mean ± SEM, *n* = 4. The statistical significance was analyzed using unpaired *t* test (* and %) or paired *t* test (@ and &). *p < 0.05 compared with the corresponding right foot (R) of the sham operation group; % *p* < 0.05 compared with the corresponding right injured foot of the surgical transection group; & *p* < 0.05 compared with the corresponding pretreatment (pre) baseline; @ *p* < 0.05 compared with the pretreatment baseline of the third mechanical ankle sprain on the 180th day (D180-pre).

Alterations in gait pattern can be seen in CAI patients. Gait parameters were analyzed using the footprint assay. Results showed a significant decrease in stance duration on the injured side in the CFL transection group (an average of 69 ± 3% decrease on the D14, D18, and D30) and the sham operation group (58% decrease on D24; 50% on D30) when compared to the corresponding pretreatment baseline (0.094 ± 0.006 s in CFL transection group and 0.116 ± 0.011 s in sham group, Figure 5A). For the mechanical sprain group, a significant decrease in stance duration was only found on the 180th day (56% reduction) when compared with the pre-treatment baseline. On the contrary, in comparison to the CFL transection group and the sham operation group, the first and second mechanical sprain induced a significantly longer stance duration on the sprained side (an average of 155 ± 7% increase on D1 and D5; 194 ± 20% on D14 to D30 compared with the CFL transection group; 168 ± 0% on D14 and D30 compared with the sham group, Figure 5A). In comparison with the baseline on the 180th day (0.065 ± 0.005 s), the third mechanical ankle sprain recurrently and significantly induced a longer stance duration (146% increase on D190 and 142% on D194, Figure 5A). In addition, the swing duration was also significantly longer in mice after the first and the second mechanical sprain than in mice treated with CFL transection (117% increase on D1 and an average of 128 ± 4% for D14 to D30, Figure 5B). However, the swing duration (Figure 5B) and step cycle (Figure 5C) in the CFL transection or the sham operation group showed no significant changes compared to the corresponding pre-treatment baseline. Moreover, the results demonstrated that the duration of step cycle was significantly longer (117% longer on D5 and 119% on D14) after the first and second mechanical sprains compared with the pre-injured baseline (0.273 ± 0.01 s) or compared with the CFL transection group (an average of 146% ± 6% longer for D1 to D30). After the third mechanical sprain, the step cycle also significantly increased on the 190th day (123% increase) compared with the baseline on the 180th day (0.224 ± 0.01 s) before the third mechanical sprain (Figure 5C). These results demonstrate that multiple mechanical sprains induce a gait change of longer step cycle due to increase in stance and swing duration.

**FIGURE 6:**
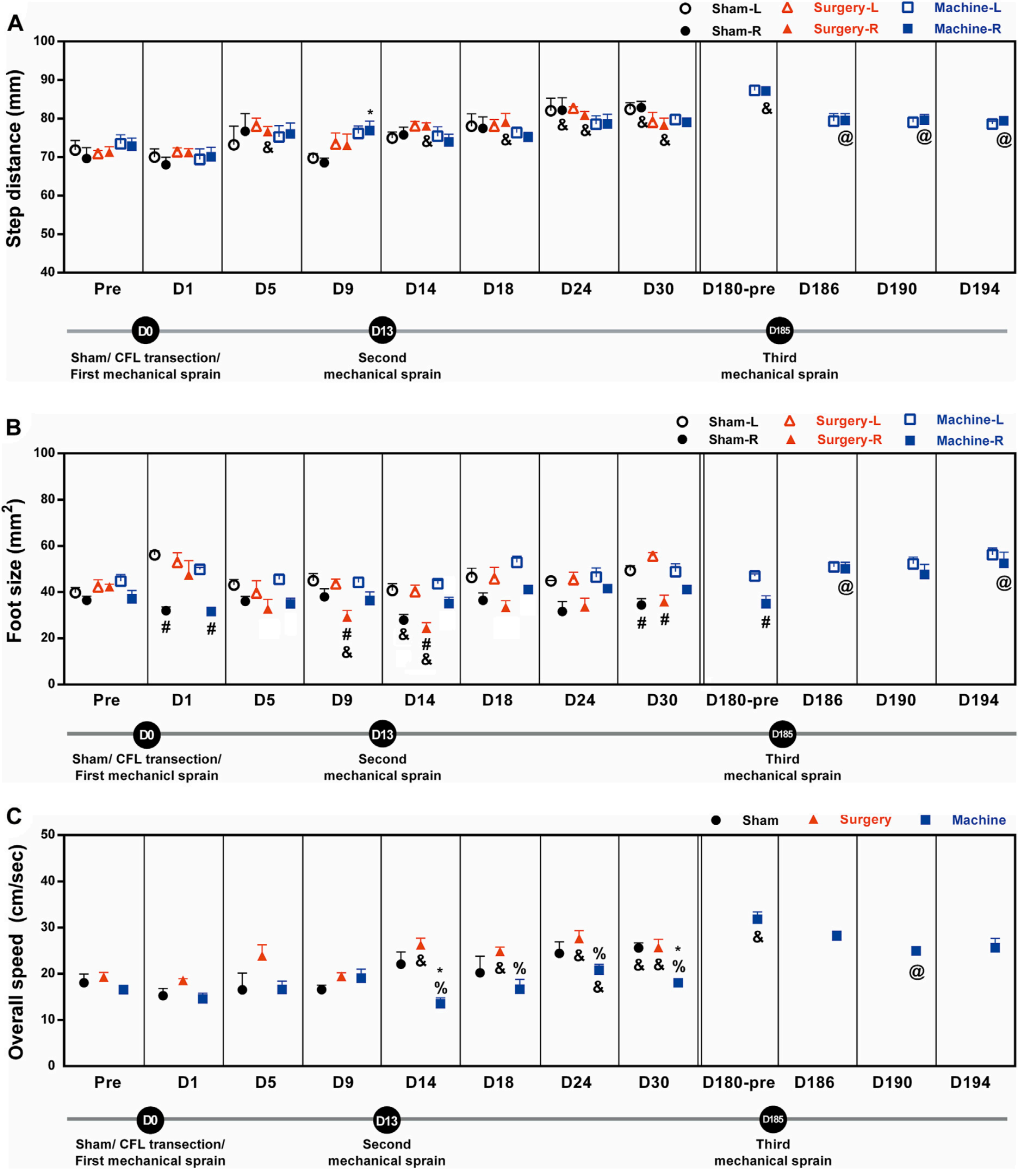
Mechanical ankle sprains induces gait abnormalities in step distance, foot size, and overall speed. Footprint was measured and analyzed on the days indicated on the graphs after sham operation, CFL transection, and three mechanical ankle sprains to obtain gait parameters including step distance **(A)**, foot size **(B)**, and overall speed **(C)**. Values are mean ± SEM, *n* = 4. The statistical significance was analyzed between groups using un-paired *t* test (* and %) or within group using paired *t* test (#, @, and &) *p < 0.05 compared with right foot (R) of sham operation group; % *p* < 0.05 compared with the right injured foot of the surgical transection group; #*p* < 0.05, a significant difference between the right injured and the left untreated foot (L) in each group; & *p* < 0.05 compared with the corresponding pretreatment (pre) baseline; @ *p* < 0.05 compared with the pretreatment baseline of the third mechanical ankle sprain on the 180th day (D180-pre).

In the mechanical sprain group, after the first and second mechanical sprains, step distance was found unaffected for the initial 30 days until a significant 120% increase on the 180th day compared with the pre-treatment baseline (72.8 ± 2.1 mm, Figure 6A). The longer step distance was also found after the CFL transection and sham operation. However, the third mechanical sprain significantly induced a decrease in step distance (an average of 91 ± 0% decrease for D186 to D194) when compared with the baseline level on the 180th day (87.2 ± 0.9 mm, Figure 6A). These results indicate that the third, but not the first and second, mechanical ankle sprain induces a gait change of shorter step length.

The foot sizes on the injured sides were significantly smaller than those on the uninjured sides after sham operation (57% reduction on D1; 64% on D30), CFL transection (an average of 64 ± 2% reduction on D9, D14, and D30), and the first mechanical sprain (64% on D1, Figure 6B). In addition, after the second mechanical sprain, the foot size was unaffected until a significant 80% reduction was found on the 180th day (D180). However, the smaller foot size on the sprained side was not found after the third mechanical sprain.

The overall walking speed increased significantly after CFL transection (an average of 135 ± 3% increase for D14 to D30) and after sham operation (142% increase on the 30th day) as compared with the corresponding pre-treatment baseline level (Figure 6C). After the first and second mechanical sprains, the overall speed was not changed for the initial 30 days until a significant 192% increase on the 180th day compared with the pretreatment baseline (16.6 ± 0.7 cm/s, Figure 6C). On the contrary, in comparison with the CFL transection or sham operation mice, the overall speed decreased significantly in mice after the second, but not the first, mechanical ankle sprain (an average of 66 ± 5% decrease vs. CFL transection for D14 to D30 and 67 ± 6% on D14 and D30 vs. sham operation). Moreover, the slow speed re-occurred after the third mechanical ankle sprain, a 78% decrease on the D190 compared with the baseline level on the 180th day (31.8 ± 1.6 cm/s, Figure 6C). These results demonstrated that the repeated mechanical ankle sprains induces a gait change of slower walking speed in contrast to faster walking speed in the CFL transection group.

## Discussion

We demonstrated a new mouse model for ankle sprain induced by mechanical wrenching of the ankle with controlled rotation angle and speed. We found that a single mechanical sprain induces ankle injury, which is similar to the effect of surgical CFL transection on ankle tenderness. Moreover, the second mechanical ankle sprain 2 weeks later induces more pronounced responses in ankle tenderness and swelling, higher beam slips, and abnormal gait. Regardless of pain and swelling, the symptoms of ankle instability, balance deficits, and abnormal gait are reproduced after the third mechanical sprain 6 months later. This mechanical sprain model can induce multiple ankle injuries and is very suitable for investigating the molecular mechanisms and treatments of ankle instability with recurrent injuries.

We first demonstrated that the ankle is twisted to 117° of inversion at a speed of 1,500° per second, two at a time. The model was able to induce an effect similar to that of the surgical CFL transection on pressure hypersensitivity and swollen ankle in mice. In addition, the ligament injury-induced hypersensitivity and swelling can be reproduced when the second mechanical ankle sprain occurs 2 weeks later; while the surgical group did not show deficits in hypersensitivity or swelling at this time point, ankle swelling was seen in the mechanical sprain mice, indicating that inflammatory edema was induced by mechanical and non-invasive ankle sprain, which is contrary to the ligament transection model that has potential additional inflammation and swelling caused by the open wound of surgery operation. The results are consistent with the swollen ankles found in humans after suffering from an ankle sprain (Delahunt et al., 2018).

In terms of the severity of ligament injury for an ankle sprain, previous studies have shown that the surgical CFL or the ATFL transection model is a grade III injury owing to the complete rupture of one or more ankle ligaments, which is different from the CAI predictor of grade II lateral ankle ligament injury (Pourkazemi et al., 2014). In the mechanical sprain model, we found that the sprain injury-induced tenderness and swelling recovered 12 and 4 days after the first and second mechanical sprain, respectively. Since ankle injuries are recoverable, the mechanical sprain condition used in the present study may be considered to induce a strain of lateral ligaments without rupture, which is known to be classified as a grade I ankle injury (Slater, 2018). Moreover, when the third mechanical sprain was performed 6 months later, it induced a delayed ankle tenderness but did not result in ankle swelling. We hypothesized that the two previous mechanical sprains may have induced laxity in the ankle ligaments, leading to ankle joint laxity; therefore the third sprain using the same twisting condition was not sufficient to result in the same notable changes in pain threshold and ankle thickness seen in the previous two sprains. Previous studies have shown in patients suffering CAI, the recurrent sprains result in unstable ankle joint due to the induction of joint laxity (Al-Mohrej and Al-Kenani, 2016). Although the hypothesis still needs to be further examined using CT scan or other histology examination methods, unstable ankle caused by loose ligaments can be supported by our results showing that significant balance defects and gait changes are found after the second and third, but not the first mechanical ankle sprain.

In the beam tests, our results demonstrate that the increased number of slips on the sprained foot are found after the first and second sprain, and persistently occurs after the third sprain, 6 months later. However, the increased foot slips in the injured ankle are only found on days 12 and 21 after CFL transection. These results are consistent with the finding that increased number of foot slips were found in the earlier periods of 3 days and 1 week after CFL transection and that mice with ATFL and CFL transection have more foot slips than sham operation and CFL transection mice (Hubbard-Turner et al., 2013; Wikstrom et al., 2015). Since persistent higher number of foot slips were found after the second and third mechanical sprain, we propose that the repeated mechanical sprains used in this study result in long-term balance defects by inducing injuries of multiple ankle ligaments. Considering the induction of balance defects as one of the major symptoms of CAI (Hertel, 2002), the results and the following abnormal gaits supports the fact that our model of recurrent mechanical ankle sprain is able to induce CAI.

The gait changes in the mechanical sprain group included a significant increase in the stance duration and step cycle (Figure 5), whereas a decrease in the step distance and overall walking speed (Figure 6), were found after each mechanical sprain as compared with the CFL transection or sham operation group. These findings further support the results that repeated mechanical ankle sprain-induced injuries of multiple ankle ligaments lead to a decrease in the step distance and walking speed because smaller step lengths and slower running speeds were found in the wheel after the transection of two ligaments (ATFL and CFL), but not limited to CFL (Hubbard-Turner et al., 2013; Hubbard-Turner et al., 2015; Wikstrom et al., 2018). Furthermore, previous results from human studies demonstrate that individuals with ankle sprains have slow walking speed and shorter step length (Punt et al., 2015). On the contrary, in both the sham operation and CFL transection groups, the stance duration decreased but the step distance and overall walking speed increased from the 14th to 30th day when compared with the pretreatment baseline. Moreover, these gait changes are also found in the mechanical sprain of mice on the 180th day, but not found in the period between the 1st and 30th day after the first and second mechanical ankle sprain. Since no footprint assay was performed in the period between the 30th day (no gait changes) and the 180th day (significant gait changes) after the first mechanical sprain, we rule out a training effect of footprint assay as the reason behind gait changes on the 180th day in the mechanical sprain group. In addition, the gait changes found in both the sham operation and CFL transection groups are comparable and also found on the 180th day in the mechanical sprain group, suggesting that the shorter stance duration, longer step distance, and faster walking speed found in all groups may be normal physiological responses. Previous results support the suggestion, showing that a longer step length is observed along with age in all the groups of sham, CFL-only, and ATFL/CFL transections (Hubbard-Turner et al., 2013; Wikstrom et al., 2015).

In addition, decreased foot size in treated side of paws was found in all groups; a persistent decrease in both sham operation and CFL transection group as well as a transient decrease in the mechanical sprain group after the first and second mechanical sprain (Figure 6B). One study demonstrated that a smaller foot print area (foot size) of the affected side of the paws was found after unilateral intracerebral hemorrhage using a similar footprint camera system used in this study (Liu et al., 2013). These findings support that the decreased foot size may be associated with the injured ankle. However, the third mechanical sprain did not induce a smaller foot size (Figure 6B) in the sprained ankle, which may be due to the ankle laxity being unable to support the foot as discussed.

The limitation of the present study is that it is hard to directly evaluate the severity of the ligamentous injuries induced by mechanical ankle sprains. For example, it is difficult to isolate the tiny CFL or ATFL and also hard to get the longitudinal section of both ligaments from the ankle samples for the examination of ligament injuries using histological staining, such as HE and collagen staining. In addition, the mechanical sprains in this study may not induce complete ligamentous tears, which is also difficult to observe using the micro-CT. One previous report demonstrated that talus displacement was still not observed from micro-CT images in the mice with CFL and ATFL transection (Liu et al., 2021). However, the limitations may be overcome by changing the experimental animals into rats and modifying the device for the rats based on the experiences of the present study. Moreover, the measurement to detect the ankle joint range of motion or mobility needs to be done urgently to elucidate the ankle laxity and functional ankle instability induced by multiple mechanical ankle sprains in the present mouse CAI model.

In conclusion, we established a new model of lateral ankle sprain using a self-designed machine with a precise control of inversion angle and speed. Our model of multiple mechanical ankle sprains can induce recurrent injuries, long-term balance deficits, and gait changes, which simulate the major characteristics of ankle instability in humans (Hertel, 2002). Moreover, the advantage of this model is that the inversion angle, speed, and times and intervals of sprains can be manipulated and controlled. Therefore, this model can be used to further study the ankle instability induced by various ankle biomechanics of sprains, which may simulate the multiform of CAI in humans. On the other hand, the mechanical sprain-induced CAI model is distinguished by surgical transection of ligaments and may also be applied for investigating the incomplete ligamentous (mild to moderate) injuries induced by CAI. This mouse model is also applied to knockout and transgenic mice to study the potential molecular mechanisms involved in ankle instability, such as nociceptors, mechanoreceptors, and proprioceptors.

## Data Availability

The raw data supporting the conclusion of this article will be made available from the corresponding author on reasonable request.
